# The Adenosine A_3_ Receptor Regulates Differentiation of Glioblastoma Stem-Like Cells to Endothelial Cells under Hypoxia

**DOI:** 10.3390/ijms19041228

**Published:** 2018-04-18

**Authors:** René Rocha, Ángelo Torres, Karina Ojeda, Daniel Uribe, Dellis Rocha, José Erices, Ignacio Niechi, Pamela Ehrenfeld, Rody San Martín, Claudia Quezada

**Affiliations:** 1 Laboratorio de Patología Molecular, Instituto de Bioquímica y Microbiología, Facultad de Ciencias, Universidad Austral de Chile, Valdivia 5090000, Chile; rrochabarrasa@gmail.com (R.R.); angelo.uach.2017@gmail.com (A.T.); kanaove@gmail.com (K.O.); daleuri@hotmail.com (D.U.); jdellis.rocha@gmail.com (D.R.); ignacioern@gmail.com (J.E.); ignacio.niechi@gmail.com (I.N.); rodysanmartin@uach.cl (R.S.M.); 2 Instituto de Anatomía, Histología y Patología, Universidad Austral de Chile, Valdivia 5090000, Chile; pamelaehrenfeld74@gmail.com

**Keywords:** glioblastoma stem-like cells, adenosine, A_3_ adenosine receptor, neovascularization, endothelial cells

## Abstract

Glioblastoma (GBM) is a neoplasm characterized by an extensive blood vessel network. Hypoxic niches of GBM can induce tumorigenic properties of a small cell subpopulation called Glioblastoma stem-like cells (GSCs) and can also increase extracellular adenosine generation which activates the A_3_ adenosine receptor (A_3_AR). Moreover, GSCs potentiates the persistent neovascularization in GBM. The aim of this study was to determine if A_3_AR blockade can reduce the vasculogenesis mediated by the differentiation of GSCs to Endothelial Cells (ECs) under hypoxia. We evaluated the expression of endothelial cell markers (CD31, CD34, CD144, and vWF) by fluorescence-activated cell sorting (FACS), and vascular endothelial growth factor (VEGF) secretion by ELISA using MRS1220 (A_3_AR antagonist) under hypoxia. We validate our results using U87MG-GSCs A_3_AR knockout (GSCs^A3-KO^). The effect of MRS1220 on blood vessel formation was evaluated in vivo using a subcutaneous GSCs-tumor model. GSCs increased extracellular adenosine production and A_3_AR expression under hypoxia. Hypoxia also increased the percentage of GSCs positive for endothelial cell markers and VEGF secretion, which was in turn prevented when using MRS1220 and in GSCs^A3-KO^. Finally, in vivo treatment with MRS1220 reduced tumor size and blood vessel formation. Blockade of A_3_AR decreases the differentiation of GSCs to ECs under hypoxia and in vivo blood vessel formation.

## 1. Introduction

Glioblastoma (GBM) is considered the most common tumor of the central nervous system and one of the most devastating types of cancer. After multimodal therapy, consisting of surgical resection followed by radio- and chemo- therapy with temozolomide, about 99% of cancer cells are eliminated; however, the tumor recurs and, as a result, patients die on average within 15 months [1,2,3]. Therapy failure is mainly attributed to a cell subpopulation called glioblastoma stem-like cells (GSCs) which, like normal neural stem cells (NSCs), have unlimited self-renewal and a multi-lineage differentiation capacity. In addition, GSCs have the potential to form in vivo tumors and exhibit a higher resistance to therapy than differentiated cancer cells [4,5,6,7]. Therefore, strategies are currently aimed at eliminating GSCs or promoting their differentiation to less aggressive phenotypes. A key feature of GBM is the presence of extensive blood vessel networks that support both tumor growth and resistance to treatment [8,9]. Unlike normal vasculature where the endothelium has a low proliferation rate, tumor vasculature is highly proliferative and the formed blood vessels are often disorganized and tortuous [10]. This entails inefficient oxygen supply and as a result, extensive hypoxic areas that promote further vasculogenesis [11]. In fact, the hypoxia inducible factor 1α (HIF-1α) induces the expression of vascular endothelial growth factor (VEGF), which in turn stimulates proliferation and migration of endothelial cells (ECs) [12,13,14,15]. GSCs are enriched in hypoxic areas within the tumor which promote both their stemness and radio- and chemo- resistance [16,17,18,19], therefore, future therapies targeting GSCs should consider this niche. In addition, like GSCs, tumor-derived ECs are found in a greater proportion in internal and hypoxic tumor regions. Importantly, GSCs can differentiate into ECs, a phenomenon where hypoxia appears to be critical [20,21,22]. However, the mechanisms and pathways that control differentiation of GSCs to ECs are not fully understood. Signaling pathways modulated by adenosine are up-regulated under hypoxia and participate in various aspects of cancer, including vasculogenesis [23,24,25,26,27]. This nucleoside is produced mainly by extracellular ATP hydrolysis and signals through its four Adenosine Receptors (ARs): A_1_ (A_1_AR), A_2A_ (A_2A_AR), A_2B_ (A_2B_AR), and A_3_ (A_3_AR) [26]. We showed that GBM cells have increased extracellular adenosine levels and exhibit high expression of A_3_AR compared to non-tumoral normal cells [28]. Similar results were observed in GSCs, where extracellular adenosine levels and A_3_AR expression are higher than in differentiated GBM cells [29,30]. However, the role of this receptor on GSCs biology is poorly understood. Therefore, our aim was to determine if A_3_AR blockade can reduce the vasculogenesis mediated by the differentiation of GSCs to ECs under hypoxia.

## 2. Results

### 2.1. Extracellular Adenosine Concentration and A_3_ Adenosine Receptor Expression Increase under Hypoxia

**Figure 1:**
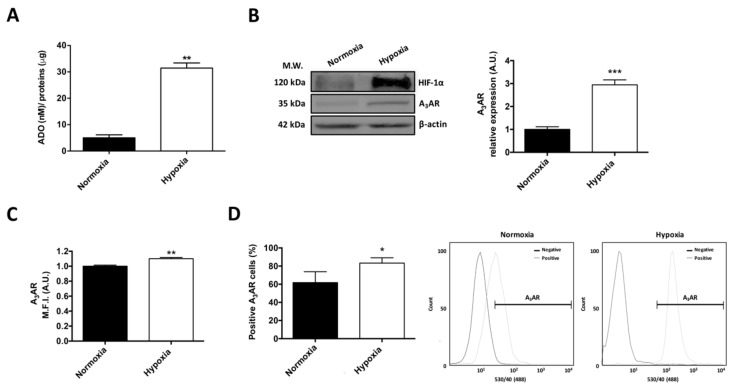
Hypoxia increases the extracellular Adenosine concentration and A_3_AR expression in glioblastoma stem-like cells. (**A**) Extracellular adenosine concentration in U87MG glioblastoma stem-like cells (GSCs) under hypoxia. U87MG GSCs were exposed to hypoxia for 24 h. Adenosine concentrations (nM) were normalized to total protein concentration (μg); (**B**) Western blot of HIF-1α and A_3_ adenosine receptor (A_3_AR) expression in U87MG GSCs under normoxia and hypoxia for 24 h; (**C**) Flow Cytometry analysis of the mean fluorescence intensity (M.F.I.) of A_3_AR expression in U87MG GSCs under normoxia and hypoxia for 24 h; (**D**) Flow Cytometry graph of A_3_AR-positive U87MG GSCs (left panel) and a representative Flow Cytometry histogram (right panel) under normoxia and hypoxia for 24 h. Graphs represent the mean ± standard deviation (S.D.). * *p* < 0.05; ** *p* < 0.01; *** *p* < 0.001 normoxia versus hypoxia (24 h). *n* = 3.

U87MG GSCs were cultured under 0.5% O_2_ in order to evaluate hypoxia effect on extracellular adenosine generation and A_3_AR expression. We found that U87MG GSCs had increased extracellular adenosine production (~7 fold) after 24 h of hypoxia (Figure 1A). A_3_AR expression increased in U87MG GSCs under hypoxia (Figure 1B,C). Similarly, the percentage of A_3_AR-positive GSCs increased under hypoxic conditions (Figure 1D). These results suggest that the high levels of extracellular adenosine in U87MG GSCs culture could activate the A_3_AR under hypoxia.

### 2.2. Differentiation of Glioblastoma Stem-Like Cells to Endothelial Cells Increases under Hypoxia

**Figure 2:**
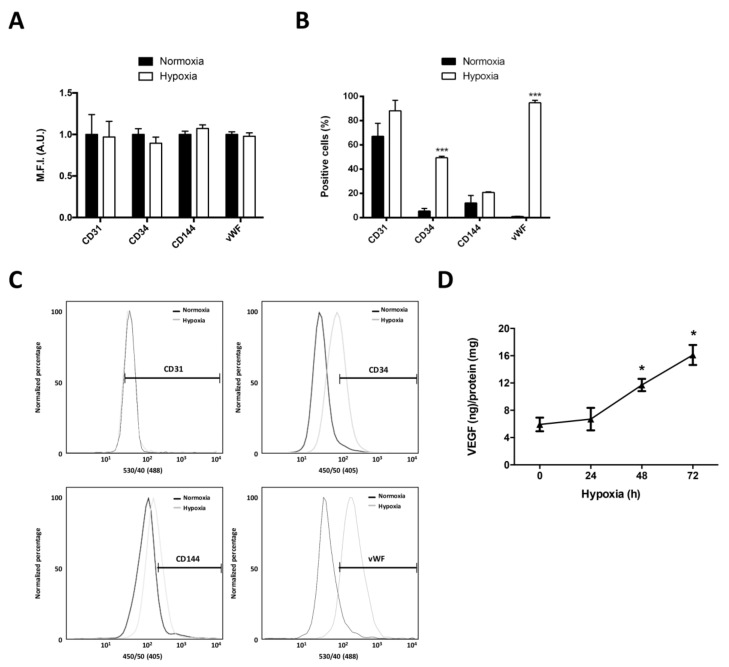
Hypoxia increases Cell Differentiation of Glioblastoma Stem-like Cells to Endothelial Cells. (**A**) Expression of Endothelial cell markers (CD31, CD34, CD144, and vWF) analyzed by Flow Cytometry using the mean fluorescence intensity (M.F.I.) in GSCs under normoxia and hypoxia (24 h); (**B**) Graphs represent the percentage of positive cells measured by Flow Cytometry for each Endothelial cell marker; (**C**) Representative Flow Cytometry histograms of (b); (**D**) VEGF-165 ELISA of the supernatant medium of U87MG GSCs in normoxia and hypoxia by 0, 24, 48 and 72 h. Graphs represent the mean ± S.D. * *p* < 0.05; *** *p* < 0.001 normoxia versus hypoxia. *n* = 3.

To evaluate the effect of hypoxia on the differentiation of GSCs to ECs we evaluated the expression of endothelial cell markers (CD31, CD34, CD144, and vWF) and VEGF secretion. No differences were observed in the expression of endothelial markers through Flow Cytometry between U87MG GSCs (Figure 2A). However, the percentage of positive cells for CD34 and vWF increased after 24 h of hypoxia (Figure 2B,C). To evaluate VEGF secretion in GSCs under hypoxia, we evaluated the presence of VEGF-165 in U87MG GSCs medium during 72 h of hypoxia. We observed an increase in VEGF-165 secretion at 48 (~2 fold) and 72 (~2.7 fold) hours under hypoxia (Figure 2D). These results suggest that U87MG GSCs could differentiate into ECs, especially under hypoxia. These results propose that hypoxia promotes the expression of endothelial cell markers and the secretion of VEGF in GSCs.

### 2.3. A_3_AR Blockade Decreases Differentiation of Glioblastoma Stem-Like Cells to Endothelial Cells under Hypoxia

**Figure 3:**
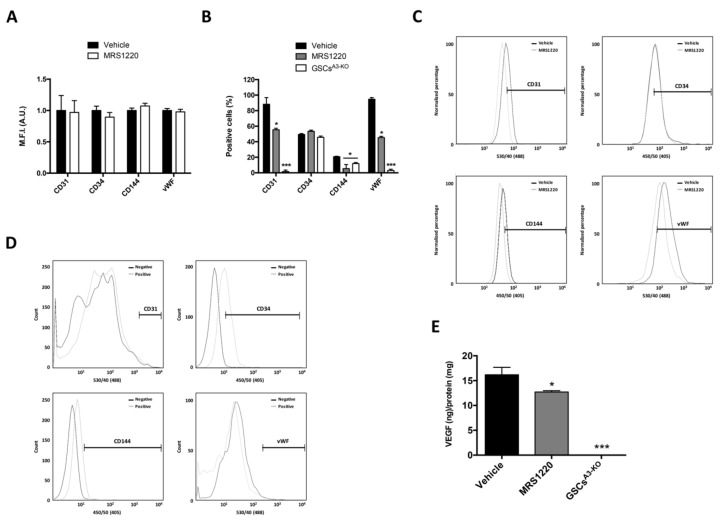
Blockade and absence of A_3_AR decreases cell differentiation of glioblastoma stem-like cells to endothelial cells under hypoxia. (**A**) Expression of endothelial cell markers (CD31, CD34, CD144, and vWF) analyzed by Flow Cytometry using the mean fluorescence intensity (M.F.I.) in U87MG GSCs treated with the selective antagonist of A_3_AR (MRS1220; 10 μM) under hypoxia (24 h); (**B**) Graphs represent the percentage of positive cells by flow cytometry for each endothelial cell marker in U87MG GSCs treated with MRS1220 (10 μM) and GSCs A_3_AR knockout (GSCs^A3-KO^) under hypoxia (24 h); (**C**) Representative flow cytometry histograms of endothelial cell markers in vehicle vs. MRS1220 treated cells; (**D**) Representative flow cytometry histograms of endothelial cell markers in GSCs^A3-KO^; (**E**) VEGF-165 ELISA of the supernatant medium of U87MG GSCs treated with 0.001% DMSO (vehicle), MRS1220 and GSCs A_3_AR knockout (GSCs^A3-KO^) under hypoxia (72 h). Graphs represent the mean ± S.D. * *p* < 0.05; *** *p* < 0.001 Vehicle was used as calibrator. *n* = 3.

We explored the effect of A_3_AR blockade on the differentiation of GSCs to ECs under hypoxia. Cells were treated with MRS1220, a selective A_3_AR antagonist, under hypoxia and then the expression of endothelial cell markers and VEGF secretion were analyzed. A_3_AR blockade did not change the expression of endothelial markers (Figure 3A), nevertheless, decreased the percentage of CD31, CD144, and vWF positive GSCs after 24 h under hypoxic conditions (Figure 3B,C). VEGF secretion in U87MG GSCs decreased ~25% with MRS1220 after 72 h of hypoxia (Figure 3E). To validate the effect of MRS1220 in U87MG GSCs differentiation to ECs, we used an A_3_AR knockout cell line (GSCs^A3-KO^) to evaluate its intrinsic differentiation ability to ECs under hypoxia. Similarly, we observed a decreased percentage of CD31, CD144, and vWF positive cells (Figure 3B,D), and an almost total decrease in VEGF secretion (Figure 3E) in GSCs^A3-KO^ under hypoxia. These results suggest that the ability of U87MG GSCs to differentiate into ECs could be regulated by A_3_AR activation under hypoxia.

### 2.4. In Vivo Antagonization of A_3_AR Decreases Tumor Size and Blood Vessel Formation

**Figure 4:**
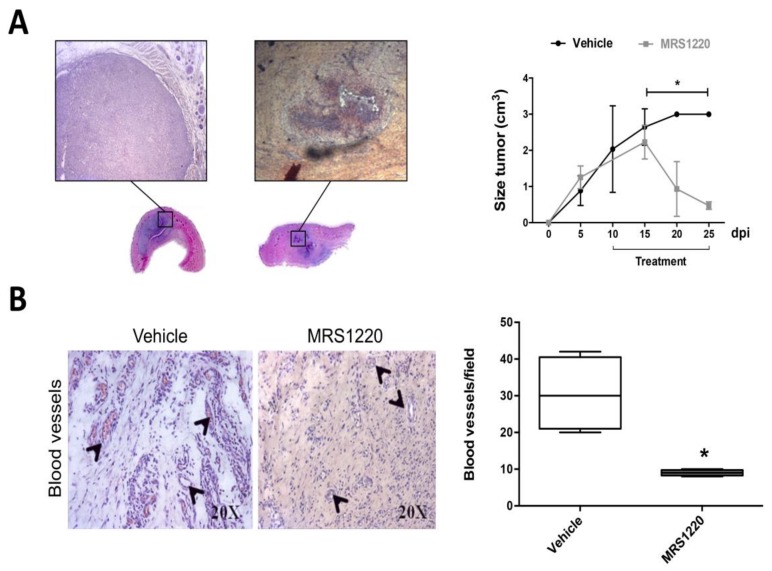
In vivo blockade of A_3_AR decreases subcutaneous tumor volume and blood vessel formation. (**A**) Subcutaneous tumors generated by C6 GSCs inoculation in Sprague–Dawley rats. Rats were treated following ten days post-inoculation (dpi) by fifteen days with vehicle (1xPBS-0.001% DMSO) and MRS1220 (0.15 mg/kg) each for 72 h. Representative sections and hematoxylin & eosin (H&E) staining of tumors treated at day twenty post-inoculation are presented (left panel). Tumor size (cm^3^) was measured each 5 days until day 25 post-inoculation (right panel); (**B**) Left panel shows the H&E histopathology analysis of tumor sections from treated rats at day twenty post-inoculation. Original magnification ×20 (H&E); Arrows indicate the location of blood vessels. Counting the amount of blood vessels per field in vehicle and MRS1220 treated groups are represented (right panel). Graphs represent the mean ± S.D. * *p* < 0.05; vehicle versus MRS1220. *n* = 3.

To ensure tumor growth, the formation of blood vessels that supply oxygen and nutrients to neoplastic cells is crucial [27]. Increased tumor volume is linked to a larger network of blood vessels, which is why in recent years the generation of new anti-angiogenic therapies has been sought [31]. To evaluate the in vivo effect of A_3_AR antagonization, we generated an allogeneic rat subcutaneous tumor using GSCs from the rat C6 glioma cell line. At day ten post-inoculation with C6 GSCs, we treated animals with MRS1220 for fifteen days. We observed a reduction close to 80% and 90% in tumor volume compared to the vehicle-treated group at day ten and fifteen post-treatment, respectively (Figure 4A). The histopathological analysis showed extensive necrotic areas with blood vessel formation, which was reverted after pharmacological blockade of A_3_AR. The number of blood vessels per field was reduced by three times with MRS1220 (Figure 4B), indicating a strong in vivo anti-angiogenic effect.

## 3. Discussion

The prognosis for GBM treatment is worsened by the presence of GSCs due to their self-renewal and cell differentiation properties, for example to endothelial cells (ECs), promoting angiogenesis and neovascularization [32]. Since GSCs are highly chemo- and radio- resistant, they are maintained in tumor niches even after treatment. This sustained maintenance and increased neovascularization is linked to the high extracellular adenosine concentrations found in tumors and even higher concentrations in hypoxic niches [27]. AR subtypes have different affinities to adenosine; A_1_ and A_2A_ are high affinity receptors and A_2B_ and A_3_ are low affinity receptors; therefore, in pathological conditions, such as cancer, extracellular adenosine levels are increased and mainly activate A_2B_AR and A_3_AR subtypes enhancing several signaling pathways, such as PI3K/AKT, MAPK, among others [27,29,33].

Knockout of ectonucleotidase CD73, which is important for extracellular adenosine production from AMP, decreased angiogenesis in melanoma models: a process that is reverted when using different AR agonists [34]. The overall effect was greater when inhibiting adenosine production compared to AR blockade, concluding that the AR subtypes have a summatory effect on angiogenesis. Several studies have confirmed that A_3_AR is important to angiogenesis in different tumors, specifically in the generation of blood vessels and neovascularization [34,35,36]. In this study, we proposed that adenosine regulates the differentiation of GSCs to ECs in vitro through A_3_AR activation, and that this promotes the formation of new blood vessels in an in vivo GBM tumor model. GSCs produce more adenosine than differentiated cells [29], and their production is enhanced under hypoxia in other tumor models [27]. In this study we showed that GSCs not only enhance adenosine production but also increase A_3_AR expression, suggesting a loop of positive regulation between the AR and its ligand; probably through expression of the transcription factor HIF-1α, which increases during early hypoxia [32]. Hypoxia-promoted cell differentiation and subsequent expression of HIF-1α have been described in different models [37], however this is poorly understood in GSCs. In this study, we showed for the first time that hypoxia increased A_3_AR expression, and its blockade decreases the cell population positive to several endothelial cell markers, such as CD34, CD144, and vWF, suggesting that GSCs could be differentiated into ECs, possibly through a mechanism dependent on extracellular adenosine-A_3_AR axis. In addition, hypoxic conditions increased VEGF secretion, which was previously observed in other tumor models but not in GSCs [38,39]. VEGF-165 secretion, which is the most abundant and potent VEGF isoform in GBM [40], was specifically evaluated. The results suggested that differentiation of GSCs into ECs could promote neovascularization and angiogenesis. These processes are highly relevant to the progression and prognosis of GBM as they support tumor growth and infiltration into surrounding healthy tissue.

In this study we used MRS1220, an A_3_AR pharmacological antagonist, to demonstrate that this receptor is involved in the differentiation of GSCs to ECs. In addition, our research group previously produced the U87MG A_3_AR KO cell line, with the ability to differentiate into GSCs, demonstrating their role in chemoresistance [29]. The A_3_AR antagonist and the KO model showed a decrease in the CD31, CD144, and vWF positive cell population, suggesting that the expression of these markers depends on AR activation; probably due to increased adenosine production in GSCs under hypoxic conditions.

Expression of in vitro markers does not necessarily reflect phenotypic changes in vivo, however, the decrease in blood vessel production in GBM models correlates with the low levels of some markers, such as CD31 [41]. To corroborate whether cell differentiation is directly linked to in vivo neovascularization, a previously validated murine model and an MRS1220 antagonist were used [29], producing a decrease in tumor size and blood vessel formation. The high concentrations of adenosine in the tumor, specifically in hypoxic niches, promotes the expression and over-activation of the A_3_AR, facilitating neovascularization. This process surely depends on HIF-1α activation; however, the possible signaling pathways involved must still be studied in depth. These results provide adenosine and its signaling with a new and important role in GBM.

## 4. Materials and Methods

### 4.1. Cell Culture

Human U87MG GBM and rat C6 glioma cell lines were acquired from the ATCC (HTB-14TM and CCL-107TM, respectively). Cells were grown in DMEM-F12 supplemented with 10% fetal bovine serum and penicillin-streptomycin (Life Technologies, Carlsbad, CA, USA). U87MG knockout for A_3_AR (GSCs^A3-KO^) were generated using the CRISPR-Cas9 protocol described by Torres et al., [29]. GSCs were obtained from U87MG and C6 cell lines using the neurosphere formation method [29]. Briefly, GSCs were cultured in Neurobasal and DMEM media for U87MG and C6 cells respectively, supplemented with 20 ng/mL bFGF, 20 ng/mL EGF, Glutamax 1X, B-27 1x, all purchased from Gibco^®^ (Thermo Fisher Scientific Inc., Waltham, MA, USA). For hypoxia experiments, GSCs were cultured in a 0.5% oxygen (O_2_) atmosphere using a hypoxia chamber (BioSpherix C-274, Oxygen sensor BioSpherix ProOx P110, Parish, NY, USA) that replaces O_2_ pumping nitrogen. GSCs cultured for 7 days were used to perform all the experiments. A_3_AR antagonizing was performed with 10 μM MRS1220 (Tocris, Park Ellisville, MI, USA) at different times depending on the experiment.

### 4.2. High Performance Liquid Chromatography (HPLC)

Quantification of adenosine production of U87MG GSCs was performed by HPLC using the protocol described by Torres et al., [29]. Briefly, GSCs were incubated in 1 mL of Tyrode’s buffer for 1 h at 37 °C. 200 µL of incubation medium was mixed with 100 µL of citrate buffer (pH 6). Adenosine, AMP, ADP, and ATP contents were quantified with 2-chloroacetaldehyde derivatizations by HPLC fractionation in a Chromolith Performance RP-18 column (Merck, Darmstadt, Germany) and by fluorescent detection [42]. Adenosine concentration (nM) was normalized to the total protein concentration (μg). 

### 4.3. Western Blot

Total protein extracts were obtained in 10 mM Tris–HCl buffer, 2% SDS, 10% glycerol, 1 mM PMSF, and protease inhibitors (Complete, Merck). Protein aliquots (50 μg) were separated by 10% SDS-PAGE followed by transfer to PVDF membranes (Bio-Rad, Hercules, CA, USA). Membranes were incubated with anti-HIF-1α (sc-10790) and anti-A_3_AR (sc-13938) antibodies (Santa Cruz Biotechnology, Dallas, TX, USA) overnight. After washing, the blots were further incubated with HRP-conjugated IgG antibody (DAKO Agilent, Santa Clara, CA, USA) for 1 h at room temperature. Finally, immune staining was visualized by using ECL plus (Amersham Pharmacia, Piscataway, NJ, USA) and the image analysis system syngene G:Box (Synoptics Ltd., Cambridge, UK). The images were analyzed by densitometry (Image J software, NIH, Bethesda, MD, USA) and each membrane was normalized to β-actin (sc-47778-HRP, Santa Cruz Biotechnology) expression.

### 4.4. Flow Cytometry

To measure endothelial cell marker expression, cells were analyzed by flow cytometry (FACS Jazz; BD Biosciences, Franklin Lakes, NJ, USA). Cells were previously fixed with PFA 3.7% for 15 min at room temperature. Cells were then blocked for 45 min (1xPBS-BSA 0.5% at room temperature) and marked with anti-CD31 (58068), anti-CD34 (562577), or anti-CD144 (561569) antibodies (BD Biosciences, Franklin Lakes). For vWF detection, cells were incubated with anti-vWF (555849) (BD Biosciences, Franklin Lakes) followed by an anti-mouse Alexa 488 (Life Technologies). Lastly, events were acquired through the FL1 filter of the cytometer.

### 4.5. Enzyme-Linked ImmunoSorbent Assay

VEGF in culture medium was quantified using Human VEGF ELISA Kit (KHG0111, Life Technologies) [40]. For kinetic analysis, a cell density of 10^4^ GSCs/well [40] were incubated for 24, 48, and 72 h under hypoxia conditions. Treatments with 10 μM MRS1220 were carried out for 72 h under hypoxia conditions with the same cell density. VEGF levels (ng) were measured according to the manufacturer’s instructions and normalized to total protein content (mg).

### 4.6. In Vivo Studies and Histopathological Analysis

A total of eight, 8 week-old male Sprague–Dawley rats were maintained under standard laboratory conditions, approved by the Ethics Committee of Animal Experiments at the Universidad Austral de Chile (Permit Number: 248-2016; date: 23 March, 2016). A density of 2 × 10^6^ GSCs C6 cells were inoculated by subcutaneous injection in previously anesthetized rats (ketamine (100 mg/kg)/xylazine (10 mg/kg) intraperitoneal). At day ten post-inoculation, animals were divided for the following treatments (i) 1xPBS-0.001% DMSO (Vehicle) (Merck), and (ii) MRS1220 (0.15 mg/kg/72 h) administered by intraperitoneal inoculation. Tumor size was measured each five days until 25 days post-inoculation when rats were euthanized by intraperitoneal administration of Sodium Thiopental (120 mg/kg). Subcutaneous tumors were removed, fixed in 3.7% paraformaldehyde, dewaxed with xylol, and rehydrated using alcohols in decreasing concentration. The samples were immersed in hematoxylin and eosin (H&E) for 5 min and finally passed through an ascending alcohol concentration followed by xylol and then mounted (Histomount, Thermo Fisher Scientific Inc.). Tumor preparations were analyzed and the blood vessel count was performed by dividing each sample into ten quadrants where the number of blood vessels was quantified and averaged using ImageJ software (NIH, Bethesda, MD, USA) [29].

### 4.7. Statistical Analysis and Artwork

Values are expressed as the mean ± Standard Deviation (S.D.), where *n* indicates number of independent experiments. Statistical analyses were performed using ANOVA, Student’s *t*-test (unpaired data), and Tukey-test. *P* values ≤ 0.05 were considered statistically significant. GraphPad Prism 6 (La Jolla, CA, USA) software was used to create all graphs and statistical analyses.

## 5. Conclusions

**Figure 5:**
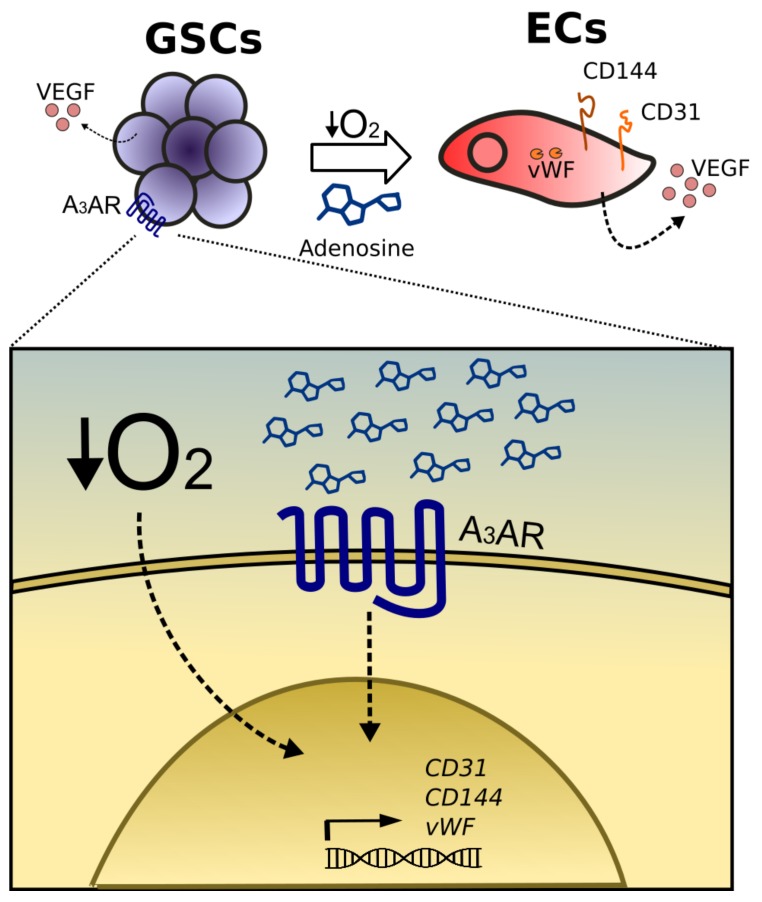
Adenosine promotes endothelial cell markers expression and VEGF secretion in GSCs under hypoxia mediated by A_3_AR activation. Under hypoxic conditions, extracellular adenosine levels and A_3_ Adenosine Receptor (A_3_AR) expression are higher than under normoxic conditions, leading to the activation of A_3_AR. Activation of A_3_AR triggers the expression of endothelial cell markers (CD31, CD34, and vWF) and VEGF production in GSCs. These processes may be mediated by the transcription factor HIF-1α.

In this study, we conclude that A_3_AR promotes GSCs differentiation to ECs under hypoxia. Expression of endothelial cells markers, such as CD144, CD31, and vWF and VEGF secretion are regulated by adenosine and A_3_AR activation. Our data suggest that hypoxic niches and the adenosine axis are responsible for neovascularization; proposing GSCs and the adenosine axis as plausible therapeutic targets for GBM (Figure 5).

## Abbreviations

GBM	Glioblastoma Multiforme
GSCs	Glioblastoma Stem-Like Cells
NSCs	Neural Stem Cells
HIF-1α	Hypoxia Inducible Factor 1
VEGF	Vascular Endothelial Growth Factor
ECs	Endothelial Cells
ARs	Adenosine Receptors
A_1_AR	A_1_ Adenosine Receptor
A_2A_AR	A_2A_ Adenosine Receptor
A_2B_AR	A_2B_ Adenosine Receptor
A_3_AR	A_3_ Adenosine Receptor
